# Always Consider the Possibility of Opioid Induced Respiratory Depression in Patients Presenting with Hypercapnic Respiratory Failure Who Fail to Improve as Expected with Appropriate Therapy

**DOI:** 10.1155/2015/562319

**Published:** 2015-03-29

**Authors:** Martin Steynor, Andrew MacDuff

**Affiliations:** Royal Stoke University Hospital, Newcastle Road, Stoke-on-Trent ST4 6QG, UK

## Abstract

Hypercapnic respiratory failure is a frequently encountered medical emergency. Two common causes are acute exacerbations of chronic obstructive pulmonary disease (COPD) and as a side effect of opioids. The two causes may coexist leading to diagnostic confusion and consequent delay in optimal management. We report a case of what was initially thought to be an exacerbation of COPD. The patient failed to improve with treatment as expected which led to the empirical administration of naloxone resulting in a dramatic reversal of her respiratory failure. The patient was subsequently discovered to be taking regular dihydrocodeine for chronic back pain.

## 1. Introduction

Hypercapnic respiratory failure is a common life threatening medical emergency. Most patients have acute exacerbations of severe COPD and the management of this has been revolutionised by the widespread adoption of noninvasive ventilation (NIV) [1]. A large number of patients also suffer from chronic pain and many take opioids prescribed by either primary or secondary care physicians [2]. This information may not be easily available when they present acutely unwell [3]. The possibility of opioid induced respiratory depression should always be considered in cases of ventilatory insufficiency as the classical features of opioid intoxication may be masked by coexisting acute pathology. We describe such a case.

## 2. Case Presentation 

A 65-year lady known to have COPD and bronchiectasis attended the emergency department complaining of a 2-hour history of increasing shortness of breath. She had previously required intubation and invasive ventilation on her last presentation one month prior to this episode. On that admission she was rapidly extubated, made an uncomplicated recovery, and had been discharged home after 48 hours. Between her episodes her exercise tolerance was approximately 100 metres. Her past medical history included chronic lower back pain and restless leg syndrome.

On examination she was haemodynamically stable. Chest auscultation revealed bilateral wheeze. Her respiratory rate was 36 breaths minute^−1^. She was alert and orientated with normal sized pupils. Chest radiograph showed hyperinflated lung fields with no focal consolidation. The full blood count revealed a leucocytosis with a neutrophil count of 19.2 × 10^9^ L^−1^.

An arterial blood gas on 6 l min^−1^ oxygen via Hudson mask (approximate FiO_2_ 0.4) demonstrated acidotic type 2 respiratory failure (pH 7.28, pCO_2_ 8.94 kPa, pO_2_ 7.03 kPa, BE 2.3 mmol L^−1^, and HCO_3_ 26.2 mmol L^−1^). The emergency physicians made a diagnosis of an acute exacerbation of COPD and appropriate medical therapy was instituted (controlled oxygen, corticosteroids, and intravenous antibiotics). The patient WAS felt to be too breathless to use an inhaler; therefore salbutamol and ipratropium were given via an air driven nebuliser. After 1 hr of treatment a repeat arterial blood gas was obtained. This showed worsening type 2 respiratory failure (pH 7.26, pCO_2_ 9.10 kPa, pO_2_ 7.34 kPa, BE 2.5 mmol L^−1^, and HCO_3_ 26.4 mmol L^−1^). The patient had also become less alert (Glasgow coma score 13) and the respiratory rate had dropped to 16. On the basis of a failure to improve with medical therapy the emergency physician decided to commence NIV in the emergency department, which the patient tolerated well without sedation and referred to the critical care team for ongoing care.

An hour later the patient was admitted to critical care and, two hours after admission, arterial blood gases were taken. These showed that the pCO_2_ had improved and was now within the normal range while maintained on noninvasive ventilation (pH 7.45, pCO_2_ 5.1 kPa, pO_2_ 9.6 kPa, BE 4.1 mmol L^−1^, and HCO_3_ 30.2 mmol L^−1^). Chest auscultation revealed minimal wheeze with good air entry throughout. Despite the significant improvement in her hypercapnia and consequent respiratory acidosis, the patient had become increasingly drowsy and her GCS had dropped from 13 on admission to critical care to 3. Spontaneous respiratory rate had dropped to 2 breaths minute^−1^. The noninvasive ventilator was delivering 12 timed breaths per minute. It was now noticed that her pupils were pinpoint. Naloxone 400 mcg was administered with immediate and dramatic effect. Her respiratory rate increased to 16 and GCS increased to 14. As the effect of the naloxone bolus wore off over the next 20 minutes the patient became more drowsy and her respiratory rate again slowed. A further bolus of naloxone was given with good therapeutic effect and a continuous naloxone infusion was commenced at 50 mcg/hr. With this therapy the patient continued to improve, her carbon dioxide level remained normal, and she did not require any further noninvasive ventilatory support. The naloxone infusion was continued for 27 hours. Reviewing the treatment charts confirmed that no opiates had been given by the paramedics or in hospital. The next morning, on questioning the patient, it transpired that she took modified release dihydrocodeine for back pain and, four days prior to admission, had increased her dose from the British National Formulary recommended maximum of 120 mg bd to 240 mg bd on her own initiative. In addition, approximately 8 hours prior to presentation, she had taken a further breakthrough dose for an exacerbation of her pain.

She made a rapid recovery and was reviewed by the pain service that altered her analgesic regimen. Spirometry on discharge showed moderately severe airflow obstruction (FEV_1_ 1.22 l, 61% predicted).

## 3. Discussion

COPD is the fourth leading cause of death [4] and one of the most common causes of hospital admissions in the UK [5]. The use of noninvasive ventilation to manage acute exacerbations has resulted in decreased mortality, a lower need for intubation, and a shorter duration of hospital stay [6]. However, there are other causes of type 2 respiratory failure and these should be borne in mind, especially if the patient is not improving on optimal treatment and/or their spirometry when well indicates only mild disease. It is important to consider other causes for alteration in mental state in patients with hypercapnic respiratory failure.

The number of opioid prescriptions in the UK is increasing [2]. Data from the US indicates that as opioid prescriptions increase so do the numbers of deaths from unintentional overdoses of these medications [7]. The situation in the UK is less clear, but data shows increased deaths related to tramadol and codeine [8]. Dihydrocodeine (DHC) is a semisynthetic opioid which is prescribed for moderate to severe pain [9]. In common with all opiates it has the potential to cause significant respiratory depression and it has been identified as one of the more common culprits in opiate related deaths [10]. Its modified release preparation is less commonly encountered but may be favoured by some patients as it provides up to 12 hours of pain relief [11]. In addition, dihydrocodeine has several active metabolites (DHC-6-O-glucuronide, dihydromorphine, dihydromorphine-3-O-glucuronide, dihydromorphine-6-O-glucuronide, and nordihydrocodeine), all of which are active at the mu opioid receptor responsible for mediating respiratory depression [12]. Some patients may therefore be susceptible to side effects of the drug many hours after ingestion. Delayed gastric emptying in the context of acute illness may exacerbate this effect [13]. Carbon dioxide retention secondary to an acute exacerbation of COPD and opiate overdose are both common causes for respiratory depression and a reduced conscious level. They may also occasionally present at the same time. However, it is unusual to for one to present after the other as seems to have occurred here. At initial presentation her symptoms seem likely to be caused by an exacerbation of her lung disease alone. The pupils were of normal size thus significant opiate intoxication at this stage improbable. However, during her treatment in the emergency department, the insidious onset of the effect of the modified release dihydrocodeine became the dominant cause of her respiratory failure. This was not quickly identified as the abnormal blood gases were ascribed to an exacerbation of her COPD while the reduction in conscious level was believed to represent carbon dioxide narcosis. This unusual sequence could have easily been missed and resulted in inappropriate treatment. Empirical administration of naloxone resulted in an immediate and dramatic improvement in the patient's respiratory rate and conscious level and we believe this strongly indicates opiate intoxication as the primary cause of her hypercapnia as opposed to the normalisation of her pCO_2_.

A respiratory rate of 12 or less in a patient who is not asleep is strongly suggestive of opiate intoxication. Furthermore since opioids reduce tidal volume before they reduce respiratory rate then significant respiratory depression can still be present with values higher than 12 [14]. We suspect this mechanism was present in this case where the patient's respiratory rate on initial assessment was elevated by the coexisting exacerbation of COPD but when her CO_2_ level was returned to normal by NIV her respiratory rate dropped as the ventilatory drive from the hypercapnia was lost. It is important to appreciate that miosis is not a universal finding in opiate intoxication [15]. The sine qua non for diagnosis of opioid induced ventilatory impairment is a prompt therapeutic effect of a trial of naloxone as was seen in this case and a trial of therapy should be given in any suspected case [16].
